# Supplementary material for: Evaluating the Harms of Cancer Testing—A Systematic Review of the Adverse Psychological Correlates of Testing for Cancer and the Effectiveness of Interventions to Mitigate These
Source: Cancers (Basel). 2023 Jun 25;15(13):3335. doi: 10.3390/cancers15133335 (PMC10340425; doi:10.3390/cancers15133335)
Supplement: Supplementary file 1 [file cancers-15-03335-s001.zip › File S1. Study characteristics for question 1.pdf]

Author, year<br>Search dates	Number of studies<br>Total number of participants	Aim	Cancer	Test
Cazacu, 2019<br>NR	7<br>872	To evaluate current knowledge about the psychological impact of routine screening for pancreatic cancer	Pancreatic	USS, MRI, blood test.
Chad-Friedman, 2017<br>1946 to 2016	22<br>11361	(1) To synthesise the evidence on distress associated with screening tests for various cancer types.<br>(2) To identify measures commonly used to measure distress at the time of cancer screening.	Breast<br>Colorectal<br>Prostate<br>Lung<br>Cervical	Mammogram, outpatient endoscopy, flexible sigmoidoscopy, colonoscopy
Metsälä, 2011<br>2000 to 2010	15<br>19906	(1) To identify factors associated with BC worry in women who require further testing following mammography.<br>(2) To explore how long BC worry persists for in these women.	Breast	Further examination of mammography screening
Montgomery, 2010<br>1983 to 2009	30<br>4746	(1) To characterise manifestations and the extent of psychological distress in women with suspected BC.<br>(2) To identify factors influencing the magnitude of psychological distress in these women.	Breast	Notification of abnormal results following mammography
Nagendiram, 2018<br>1991 to 2018	13<br>872	(1) To complete a systematic literature review of the factors that prevent Australian women from participating in cervical screening.<br>(2) To identify factors hindering attendance at cervical screening in Australian women.	Cervical	HPV testing
Nelson, 2016<br>NR	59	To review studies reporting on false positive results, overdiagnosis, anxiety, pain and radiation exposure in average risk women following mammography, MRI, or USS.	Breast	Mammography, MRI, USS

	33532			
O'Connor, 2016<br><br>1986 to 2014	23<br><br>4489	(1) To identify adverse psychological outcomes of colposcopy and predictors for the latter.<br>(2) To explore how these outcomes progress over time.	Cervical	Colposcopy
van der Velde, 2017<br>R	7<br><br>3036	To improve our understanding of the psychological impact of a false positive CRC screening result and suggest recommendations for primary care.	Colorectal	Colonoscopy
Wu, 2016<br>NR	13<br><br>9416	(1) To summarize the current state of the scientific knowledge on psychological burden associated with LCS.<br>(2) To evaluate current knowledge of the psychological cost of LCS.	Lung	Low Dose CT scan (LDCT)
Yang, 2018<br>2005 to 2017	58<br><br>24490	(1) To assess the extent of anxiety prior to colonoscopy or FS.<br>(2) To explore concerns related to colonoscopy or FS.<br>(3) To identify predictors of this anxiety.<br>(4) To determine which interventions are helpful in mitigating this anxiety.	Colorectal	Colonoscopy, Flexible sigmoidoscopy

†Abbreviations: BC – breast cancer; CRC – colorectal cancer; FS – flexible sigmoidoscopy; HIV – Human Immunodeficiency Virus; HPV – Human Papilloma Virus; LCS – lung cancer screening; LDCT – low dose computed tomography; MRI – Magnetic Resonance Imaging; NCSP – National Cancer Screening Programme; RCT – Randomised Controlled Trial; USS - ultrasonography

Table S1.1. Study characteristics of systematic reviews assessing variables associated with psychological morbidity in individuals undergoing either screening or diagnostic testing for any cancer (Question 1)

Author	Country, year	Type of study N participants	Aim	Type of cancer	Test
Al-Alawi	Oman, 2019	Cross-sectional N=300	To assess the physical and psychosocial impact associated with a referral for cancer screening and identify the associated risk factors for anxiety.	Breast	Mammography
April-Sanders	USA, 2018	Cross-sectional N=230	To examine the magnitude and risk factors for BC worry in Hispanic and migrant populations.	Breast	Mammography
Bekkers	Netherlands, 2002	Cross-sectional N=47	To reduce anxiety associated with cervical cancer screening by identifying its causes, predictors, and manifestations.	Cervix	Colposcopy
Bolejko	Sweden, 2015	Cross-sectional N=399	To evaluate the predictors and psychosocial impact of a FP result as part of BC screening.	Breast	Mammography
Drolet	Canada 2011	Cross-sectional N=942	To assess the quality of life in women following an abnormal smear result and identify predictors of negative psychosocial outcomes in this population.	Cervix	Notification of abnormal smear result
El Hachem	Lebanon 2019	Cross-sectional N=100	To evaluate the psychosocial impact of benign breast biopsies on Lebanese women after a screening mammography and the effect of these biopsies on patients’ attitudes toward subsequent screening.	Breast	Mammography

French	UK<br>2006	Cross-sectional<br><br>N=427	To assess whether anxiety levels persist in women with an inadequate smear and who subsequently have a normal result and identify predictors of raised levels of distress.	Cervix	Abnormal cervical smear result
Gray	UK<br>2006	Cross-sectional<br><br>N=3671	To define the extent of anxiety and depression following a low-grade abnormal smear result, identify risk factors for higher levels of anxiety, and assess whether certain subgroups of women are particularly at risk of psychosocial morbidity.	Cervix	Notification of abnormal smear result
Hilal	Germany<br>2017	RCT<br><br>N=225	To examine whether video colposcopy is effective at reducing anxiety during colposcopy.	Cervix	Colposcopy
Kola	Ireland<br>2012	Cross-sectional<br><br>N=164	To identify psychosocial factors that predict distress in first time attenders at colposcopy.	Cervix	Colposcopy
Liao	Taiwan<br>2008	Cross-sectional<br><br>N=153	(1) To explore whether uncertainty and anxiety levels vary in women with suspected BC during the diagnostic period.<br>(2) To compare anxiety and uncertainty levels between women with benign and malignant results during the diagnostic period.<br>(3) To identify predictors of anxiety.	Breast	Breast biopsy
Maissi	UK<br>2004	Cross-sectional<br><br>N=1376	To assess the psychosocial impact of testing for HPV following a borderline or mildly dyskaryotic smear.	Cervix	HPV testing

Medd	Australia 2005	Cross-sectional N=31	To explore men's experience of prostate biopsy and assess whether a trial of interventions to mitigate distress is feasible.	Prostate	Prostate needle biopsy
O'Connor	Ireland 2016	Cross-sectional N=584	To assess how levels of anxiety and colposcopy associated worries vary at 4, 8 and 12 months and identify predictors for these adverse outcomes.	Cervix	Colposcopy
Wiggins	USA 2017	Cross-sectional N=375	To explore how demographic, clinical and socio-environmental factors influence psychological responses to a false positive result for OC in asymptomatic average risk women attending for screening.	Ovary	Transvaginal USS
Wiggins	USA 2019	Cross-sectional N=373	To explore how cancer-specific distress varies with time following a false positive OC screening result.	Ovary	Transvaginal USS

†Abbreviations: BC – breast cancer; FP – false positive; HPV – Human Papilloma Virus; OC – ovarian cancer; RCT – Randomised Controlled Trials; USS - ultrasonography

Table S1.2. Study characteristics of cross-sectional studies assessing variables associated with psychological morbidity in individuals undergoing either screening or diagnostic testing for any cancer (Question 1)
