# Supplementary material for: Evaluating the Harms of Cancer Testing—A Systematic Review of the Adverse Psychological Correlates of Testing for Cancer and the Effectiveness of Interventions to Mitigate These
Source: Cancers (Basel). 2023 Jun 25;15(13):3335. doi: 10.3390/cancers15133335 (PMC10340425; doi:10.3390/cancers15133335)
Supplement: Supplementary file 1 [file cancers-15-03335-s001.zip › File S2. Study characteristics for question 2.pdf]

Author, Country, year<br><br>Type of study	Aim	Outcomes	Type of cancer	Test	Eligibility criteria	Demographics	
Camail Cameroon, 2019<br><br>Two-arm parallel group RCT	To compare anxiety levels in women undergoing a visual inspection of cervix whilst watching the procedure and those who do not.	Primary:<br>1. Procedure-related anxiety	Cervix	Visual inspection of cervix	Setting:<br>Outpatient clinic in low resource area.<br><br>Inclusion:<br>1. Women aged 30 to 49 years.<br>2. Cervical cancer screening programme only.<br>3. Informed consent.<br><br>Exclusion:<br>1. Women unable to comply with the study protocol.	Age, mean (SD) years<br><br>Education (%)<br><br><br><br><br><br><br><br>Employment (%)<br><br><br><br>Ethnicity<br><br>Gender (%)	39.1 (5.2)<br><br>I: High school (63), Elementary school (18), University (12)<br><br>C: Apprenticeship (56), High school (22), None (10)<br><br><br>Similar for I and C: Employed (65), Housewife (26).<br><br>Not reported<br><br>Female (100)
Chantawong Thailand, 2017<br><br>Two-arm parallel group RCT	To compare pain, anxiety, and satisfaction between women, who listened to music, and those who did not during loop electrosurgical excision	Primary:<br>1. Procedure related pain<br><br>Secondary:<br>1. Procedure-related anxiety<br>2. Procedure-related satisfaction	Cervix	Loop electrosurgical excision procedure (LEEP)	Setting:<br>Outpatient clinic<br><br>Inclusion:<br>1. Adult women with confirmed or suspected high-grade changes of cervix.<br><br>Exclusion:	Age, median (range) years<br><br>Education<br><br>Ethnicity<br><br>Gender (%)	I: 46.5 (25 to 74)<br>C: 44.0 (25 to 63)<br><br><br>NR<br><br>NR<br><br>Female (100)

	procedure (LEEP).				1. Allergy to lidocaine<br>2. Pregnant<br>3. Previous major surgery to cervix or uterus<br>4. Cardiac pacemaker or known cardiac arrhythmia<br>5. Neurological conditions affecting perception of pain.<br>6. Previous lower urinary tract cancer<br>7. Coagulation disorders<br>8. History of drug dependence<br>9. Lower genital tract infection<br>10. Suspected cancer of cervix<br>11. Hearing difficulty		
Chlan<br>USA, 2000<br><br>Two-arm parallel group RCT	To assess the effectiveness of music therapy on anxiety, discomfort, satisfaction and compliance in patients having FS.	Primary:<br>1. Procedure-related anxiety<br><br>Secondary:<br>1. Procedure-related discomfort<br>2. Patient satisfaction<br>3. Adherence with screening guidelines	Colon	Flexible sigmoidoscopy	Setting:<br>Single tertiary centre<br><br>Inclusion:<br>1. Adult male and female<br>2. Screening FS<br>3. English as primary language<br>4. Minimal hearing impairment<br>5. Mentally competent	Age, mean (SD) years<br><br>Education<br><br>Ethnicity (%)<br><br><br><br>Gender (%)	54.6 (11.5)<br><br>NR<br><br>White (96.8), African American (1.6), Hispanic (1.6)<br><br>Female (69), male (31)

Cruickshank UK, 2005<br><br>Two-arm parallel group RCT	To compare the effect of self-administered isoflurane and desflurane on women's experience of outpatient treatment at colposcopy.	Primary:<br>1. Procedure related pain, anxiety, and satisfaction.<br><br>Secondary:<br>1. Default to follow up after treatment.	Cervix	Colposcopy	Setting:<br>Colposcopy clinic serving regional population<br><br>Inclusion:<br>Women attending for large loop excision of transformation zone (LLETZ) for cervical intraepithelial neoplasia (CIN)<br><br>Exclusion:<br>1. No treatment required.<br>2. Pregnant<br>3. Currently taking a monoamine–oxidase inhibitor<br>4. Had to drive home from the clinic themselves.	Age, mean (SD) years: I: 32.71 (9.78); C: 31.53 (9.12)<br><br>Education: NR<br><br>Ethnicity: NR<br><br>Gender (%): Female (100)
de Bie Netherlands, 2011<br><br>Two-arm parallel group RCT	To assess whether provision of targeted information (by mail or phone) mitigates anxiety in women attending for colposcopy.	Primary outcome:<br>1. Procedure-related anxiety	Cervix	Colposcopy	Setting:<br>Colposcopy clinic<br><br>Inclusion:<br>Adult women with abnormal smear results.<br><br>Exclusion:<br>1. Previous colposcopy<br>2. Unable to attend 15 minutes before the	Age, median (IQR) years: 34.0 (30-40)<br><br>Education (%): At least college (47.3)<br><br>Ethnicity: NR<br><br>Gender (%): Female (100)

					scheduled appointment<br>3. Not fluent in Dutch		
Dey<br>UK, 2002<br><br>Two-arm parallel group RCT	To determine the cost to the NHS and the impact on anxiety of a one stop clinic for assessing women with suspected BC.	Primary:<br>1. Procedure-related anxiety<br>2. Mean cost per patient	Breast	Mammography, USS, aspiration cytology, same-day results and management plan.	Setting:<br>Teaching hospital<br><br>Inclusion:<br>Women with a breast lump and aged 35 or over	Age, mean (SD) range years<br><br>Gender (%)	I: 50 (10.5), range 35-86<br>C: 49 (10.5), 35-95<br><br>Female (100)
Domar<br>USA, 2005<br><br>Three-arm parallel group RCT	To examine the effect of a relaxation audiotape pre- and during mammography on pain and anxiety levels.	Primary:<br>1. Procedure-related pain and anxiety	Breast	Mammography	Setting:<br>Outpatient screening mammography facility in tertiary care teaching hospital<br><br>Inclusion:<br>Women attending screening mammography only.<br><br>Exclusion:<br>1. Not fluent in English<br>2. Women intending to listen to own audtiotape.<br>3. Women who had taken pain or anxiety medication prior to their procedure, or those with an existing psychiatric condition.<br>4. History of BC	Age, mean years<br><br>Education (%)<br><br>Ethnicity (%)<br><br>Gender (%)	51.7<br><br>More than high school (73-92)<br><br>White (78-91), Black (7-13), other (2-10)<br><br>Female (100)

Ferrante<br>USA, 2008<br><br>Two-arm parallel group RCT	To assess the effectiveness of a patient navigator on time to diagnosis, anxiety levels, and satisfaction after an abnormal mammogram.	Primary:<br>1. Diagnostic interval<br>2. Procedure-related anxiety<br>3. Patient satisfaction.	Breast	Mammography	Setting:<br>Urban university hospital serving low income minority population.<br><br>Inclusion:<br>Women with suspicious mammogram results.<br><br>Exclusion:<br>1. Women under age 21<br>2. Not fluent in English.	Age<br>mean (SD) years<br><br>Education (%)<br><br><br>Employment (%)<br><br><br>Ethnicity (%)<br><br><br>Gender	50.1(11.6)<br><br><br>High school and less (76.2), College or more (23.7)<br><br>Unemployed (65.7), Employed (34.4)<br><br>Black (59), Hispanic (27.6), other (13.3)<br><br>Female (100)
Hersch<br>Australia, 2006<br><br>Two-arm parallel group RCT	To assess whether the addition of information on overdetection improved informed choice about breast screening	Primary:<br>1. Informed choice about breast screening<br><br>Secondary:<br>1. Decisional conflict<br>2. Confidence in decision making<br>3. Procedure-related anxiety<br>4. Worry about BC<br>5. Anticipated regret (later regret if do not screen)	Breast	Mammography	Setting:<br>New South Wales<br><br>Inclusion:<br>women aged 48-50 years<br><br>Exclusion:<br>1. Mammogram in the past 2 years<br>2. Previous history of BC<br>3. increased risk of BC e.g., strong family history<br>4. Language barrier.	Age<br>mean (SD) years<br><br>Country of birth (%)<br><br>Education (%)<br><br><br><br><br><br><br>Ethnicity<br><br>Gender (%)	49.7 (0.4)<br><br><br>Australia or NZ (80), abroad (20)<br><br>Trade certificate or diploma (41-46), degree or graduate diploma/certificate (28-29), intermediate school certificate or less (15-17), higher school certificate (10-13).<br><br>NR<br><br>Female (100)

		6. Anticipated regret (later regret if do screen)<br>7. Temporal orientation<br>8. In deciding whether to have screening, how important is it for patients to consider the chance of (1) avoiding death from BC; (2) overdetection; (3) false positives<br>9. Perceived risk of BC<br>10. Perceived risk BC relative to average women<br>11. Compared with the average screened woman, if patients are screened how likely is it that they would (1)				

		avoid dying from BC; (2) experience overdetection					
Hilal Germany, 2017<br><br>Two-arm parallel group RCT	To examine whether video colposcopy reduces anxiety in patients having colposcopy.	Primary:<br>1. Procedure-related state anxiety<br><br>Secondary:<br>1. Procedure-related pain<br>2. General unpleasantness<br>3. Anxiety during colposcopy<br>4. Satisfaction with information 5. Overall satisfaction	Cervix	Colposcopy	Setting:<br>Hospital and doctor's office<br><br>Inclusion:<br>1. 18 to 80 years<br>2. Women referred due to cervical abnormalities<br>3. First time attenders to colposcopy<br><br>Exclusion:<br>1. Pregnant<br>2. Language barrier<br>3. Known anxiety disorder or depression<br>4. Previous treatments<br>5. Known cancer (any).<br>6. Inadequate colposcopy	Age<br>mean (SD) years<br><br>Education level*<br><br><br>Ethnicity<br><br>Gender<br><br>*range 1–8 (1=minimum education required by law, 8=postgraduate education)*	I: 36.1(9.5)<br>C: 35.5 (10.8)<br><br>I: 4 (3–5)<br>C: 3 (2–6)<br><br>NR<br><br>100% female
Hilal Germany, 2018<br><br>Two-arm parallel group RCT	To assess whether music reduces anxiety in patients having colposcopy.	Primary:<br>1. Procedure-related anxiety<br><br>Secondary:<br>1. Reduction of heart rate<br>2. Procedure-	Cervix	Colposcopy	Setting:<br>Hospital and doctor's office<br><br>Inclusion:<br>1. 18 to 80 years<br>2. Women referred due to cervical abnormalities	Age,<br>mean (SD) years<br><br>Educational level*<br><br><br>Ethnicity (%)	I: 36.1(9.5)<br>C: 35.5 (10.8)<br><br>I: 3 (2–3)<br>C: 3 (2–4)<br><br>German (85-92),<br>Mediterranean (5-8),

		related pain<br>3. General unpleasantness<br>4. Anxiety during colposcopy<br>5. Overall satisfaction			3. First time attenders to colposcopy<br><br>Exclusion:<br>1. Pregnant<br>2. Language barrier<br>3. Known anxiety disorder or depression<br>4. Previous treatments<br>5. Known cancer (any).<br>6. Inadequate colposcopy	 <br>Gender (%)<br><br>**range 1–8 (1=minimum education required by law, 8=postgraduate education)*	Eastern EU (4-7), Asian (2)<br><br>Female (100)
Lang<br>USA, 2006<br><br>Three-arm parallel group RCT	To examine whether self-hypnotic relaxation could reduce pain and anxiety levels in patients undergoing large core needle biopsy.	Primary: 1. Procedure-related pain and anxiety	Breast	Large core needle biopsy	Setting:<br>Urban tertiary university-affiliated medical centre<br><br>Inclusion:<br>1. Adult male and female<br><br>Exclusion:<br>1. Unable to give informed consent or pass screening for impaired mental function or psychosis.<br>2. Unable to hear or understand English.	Age, median (range) years<br><br>Ethnicity (%)<br><br>Gender	50 (18 –82)<br><br>Caucasian (>70)<br><br>Female (100)
Shaik<br>USA, 2010<br><br>Two-arm parallel group RCT	To assess whether an educational pamphlet lowers anxiety levels before colonoscopy, and	Primary:<br>1. Procedure-related anxiety<br><br>Secondary:<br>1. Quality of	Colorectal	Colonoscopy	Setting:<br>Local clinic affiliated to tertiary referral centre.<br><br>Inclusion:<br>Average-risk or high-risk	Age, mean years<br><br>Education<br><br>Ethnicity	58<br><br>NR<br><br>NR

	if its use affects the quality of the prep or the amount of medication used during the procedure.	bowel preparation<br>2. Amount of sedative medications used.			screening colonoscopy for the first time i.e., average-risk patients who are asymptomatic and over the age of 50, and younger patients with risk factors CRC<br><br>Exclusion:<br>1. Previous colonoscopy<br>2. Not fluent in English or Spanish<br>3. Pregnant<br>4. Severe cognitive impairment or learning disability<br>5. On anxiolytics<br>6. Unable to give informed consent	Gender (%)	Female (60), male (40)

†Abbreviations include BC – breast cancer; CIN – cervical intraepithelial neoplasia; CRC – colorectal cancer; FOB – faecal occult blood; FS – flexible sigmoidoscopy; LCS – lung cancer screening; LDCT – low dose computed tomography; LEEP - Loop electrosurgical excision procedure; LLETZ – large loop excision of transformation zone; NHS – National Health Service; USS – ultrasonography

Table S2. Study characteristics of RCTs investigating the effectiveness of interventions for mitigating anxiety associated with cancer testing (Question 2)
