# Supplementary material for: Evaluating the Harms of Cancer Testing—A Systematic Review of the Adverse Psychological Correlates of Testing for Cancer and the Effectiveness of Interventions to Mitigate These
Source: Cancers (Basel). 2023 Jun 25;15(13):3335. doi: 10.3390/cancers15133335 (PMC10340425; doi:10.3390/cancers15133335)
Supplement: Supplementary file 1 [file cancers-15-03335-s001.zip › File S3. Variables associated with psychological morbidity identified from cross-sectional studies for question 1.pdf]

Author, year	Measurement tool	Variables	Results	
Al-Alawi, 2019	Psychological Capital Questionnaire (PCQ)			**Mean ± SD, p-value**
		<u>Age</u>		
		- Social score	40-50	1.69 ± 2.24, p=0.008
			50-60	0.94 ± 1.67
			60	0.61 ± 1.16
		- Emotional score	p=0.189	
		- Physical score	p=0.182	
		<u>Educational level</u>		Categories include illiterate, literate, secondary school and university graduate
		- Emotional score	p=0.553	
		- Physical score	p=0.106	
		- Social score	0=0.405	
		<u>Employment status</u>		
		- Emotional score	Employed	4.17 ± 4.11, p=0.043
			Unemployed	3.26 ± 3.76
		- Social score		1.67 ± 2.23, p=0.012
			Employed	1.13 ± 1.88
			Unemployed	
		- Physical score		
		<u>Family history of cancer</u>	p=0.637	
				1.67 ± 2.23, p=0.047
		- Emotional score		1.13 ± 1.88
			Yes	
			No	1.67 ± 2.23, p=0.008
		- Physical score		1.13 ± 1.88
			Yes	

		- Social score	No 1.67 ± 2.23, p=0.007<br>1.13 ± 1.88<br>Yes<br>No
April-Sanders, 2018	Self-designed questionnaire	 <br>Age at interview<br><br>Perceived Absolute Risk<br><br>Perceived Comparative Risk (Same/More risk compared to Lower Risk)	**High vs Low/Moderate Breast Cancer Worry, OR (CI)**<br><br>0.95 (0.90-1.00)<br><br>1.66 (1.28 – 2.14)<br><br>2.73 (1.23 – 6.06)
Bekkers, 2002	Dutch version of STAI	 <br>Experienced waiting time as short vs long<br><br>Patient has a partner vs no partner<br><br>Satisfied with info from GP vs not satisfied<br><br>Satisfied with info from gynaecologist etc<br><br>Colposcopy was clearly explained	**STAI score (SD)**<br><br>45.5 (12.9) vs 57.1(9.7), p<0.01<br><br>45.3 (11.2) vs 65.0 (11.), p<0.001<br><br>42.7 (12.2) vs 55.2 (12.6), p<0.05<br><br>38.9 (12.7) vs 50.3 (7.0), p<0.05<br><br>38.6 (10.0) vs 53.2 (9.8), p<0.05

		Age <40<br>Education less than college<br>Cervical smear mild dyskaryosis or less<br>Cancer among relatives<br>Patient has children	NS (figures not reported)<br>NS<br><br>NS<br><br>NS<br><br>NS
Bolejko, 2015	Swedish Consequences of Screening in Breast Cancer (COS-BC)	 <u>Worry</u><br>- Anxiety<br><br><u>Susceptibility</u><br>- Anxiety<br>- Sense of dejection<br>- Behavioural<br><br><u>Lack of social support</u><br>- Behavioural<br>- Sleep<br><br><u>Dissatisfied with own knowledge about BC</u><br>- Sense of dejection<br>- Existential values<br><br><u>Dissatisfied with information at recall</u><br>- Sense of dejection<br>- Anxiety<br>- Behavioural<br>- Sleep	**OR (95% CI), p-value**<br><br>1.20 (1.06-1.37), p=0.005<br><br><br>1.53 (1.21-1.95), p<0.001<br>1.49 (1.20-1.84), p<0.001<br>1.50 (1.21-1.87), p<0.001<br><br><br>1.15 (1.03-1.29), p=0.016<br>1.20 (1.06-1.35), p=0.003<br><br><br><br>2.08 (1.02–4.26), p=0.045<br>3.11 (1.45–6.67), p=0.004<br><br><br><br>2.28 (1.05–4.95), p=0.037<br>2.56 (1.17–5.61), p=0.019<br>2.42 (1.12–5.24), p=0.025<br>2.38 (1.09–5.24), p=0.031

		Early recall<br>- Sense of dejection<br>- Anxiety<br>- Behavioural<br>- Sleep<br><br>Country of origin (other vs Sweden)<br>- Anxiety<br>- Behavioural<br>- Sleep<br><br>Level of education (lower vs higher)<br><br>Age	 10.31 (5.01–21.23), p<0.001<br>6.25 (3.16–12.38), p<0.001<br>3.21 (1.68–6.14), p<0.001<br>5.24 (2.72–10.07), p<0.001<br><br><br>2.40 (1.11–5.19), p=0.026<br>2.96 (1.36–6.45), p=0.006<br>3.71 (1.62–8.51) p=0.002<br><br>2.89 (1.15–7.27), p=0.024<br><br>P=NS
Drolet, 2011	STAI	 <br>Univariate analysis<br>Age<br><br>Education<br>- Elementary/high school<br>- College<br>- University<br><br>Living<br>- Alone<br>- With partner<br>- With others	**Difference in STAI score of women with an abnormal smear result compared with women with a normal smear result., p-value (95% CI)**<br><br>P=NS<br><br>p=0.02<br>5.2 (2.4-7.9)<br>9.9 (7.5–12.3)<br>9.8 (7.2–12.4)<br><br>p=0.03<br>11.9 (8.3–15.4)<br>6.8 (4.8–8.8)<br>9.7 (6.9–12.4)

		Household income	p=0.004
		- <$20,000	9.5 (6.0–12.9)
		- $20,000–$49,999	11.2 (8.5–13.9)
		- $50,000–$79,999	6.8 (3.5–10.1)
		- ≥$80,000	3.9 (0.7–7.2)
		Number of stressful life events	p=0.04
		- 0	7.0 (4.8–9.2)
		- 1	8.8 (6.5–11.1)
		- ≥2	12.8 (8.9–16.7)
		Smoking status	p=0.004
		- Current	11.8(9.1–14.5)
		- Former	9.2(6.4–11.9)
		- Never	5.9(3.7–8.2)
		Stable relationship	P=NS
		Number of lifetime partners	p= 0.04
		- 1–10	7.6(5.8–9.3)
		- ≥11	11.2(8.1–14.4)
		Screening history prior to qualifying abnormal smear	p=0.05
		- No history of abnormal smear	9.8 (7.8–11.8)
		- Prior smear was abnormal	8.9 (5.9–11.9)
		- Prior smear was	5.2 (2.1–8.3)

		normal, but already had an abnormal smear in the past<br><br>Severity of abnormal smear<br><br>Communication of the result<br>- In person<br>- By telephone/letter<br><br>Woman's reported understanding of the result<br>- Not well<br>- Well<br>- Very well<br><br>Perceived risk of cancer compared with other women<br>- Lower<br>- Same<br>- Higher<br>- Do not know<br><br><u>Multivariate analysis</u><br>Household income<br>- <$20,000<br>- $20,000–$49,999<br>- $50,000–$79,999	 <br><br><br>P=NS<br><br><br>p=0.02<br><br>9.9 (8.1–11.8)<br>6.2 (3.8–8.6)<br><br>p=0.003<br><br><br>14.2 (10.5–18.0)<br>8.7 (6.1–11.2)<br>6.8 (4.8–8.8)<br><br>p=0.001<br><br><br>6.0 (1.5–10.6)<br>4.6 (1.6–7.6)<br>11.5 (9.2–13.8)<br>8.5 (5.6–11.3)<br><br><br>p=0.03<br>8.7 (5.3–12.2)<br>1.1 (8.5–13.7)

		- ≥$80,000<br><br>Smoking status<br>- Current<br>- Former<br>- Never<br><br>Communication of the result<br>- In person<br>- By telephone/letter<br><br><br>Woman's reported understanding of the result<br>- Not well<br>- Well<br>- Very well<br><br>Perceived risk of cancer compared with other women<br>- Lower<br>- Same<br>- Higher	7.1 (3.9–10.2)<br><br>5.0 (1.9–8.2)<br><br>p=0.03<br>10.8 (8.1–13.5)<br>9.4 (6.7–12.1)<br>6.4 (4.2–8.7)<br><br>p=0.01<br><br>10.1 (8.2–11.9)<br>6.1 (3.7–8.4)<br><br><br>p=0.002<br><br><br>14.5 (10.7–18.2)<br>8.7 (6.2–11.3)<br>6.8 (4.8–8.7)<br><br>p=0.01<br><br><br>7.5 (3.3–11.7)<br>5.6 (2.8–8.4)<br>10.9 (8.7–13.0)
El Hachem, 2019	Negative PCQ; self-designed questionnaire in Arabic	 Age<br><br>Religion(Christian, Druze, Muslim)	**mean (SD), p-value**<br>p=NR<br><br>p=NR

		Result of the last mammography<br>- Suspicious<br>- Benign<br>- Normal<br><br>Number of biopsies<br>- 1<br>- 2<br>- 3<br>- 4<br>- 5<br>- 8<br><br>Date of the last biopsy<br>- 1st 50%<br>- 2nd 50%<br><br>Worry about BC<br>- Frequently<br>- Often<br>- Rarely<br>- Never<br><br>Worry affects humour<br>- Frequently<br>- Often<br>- Rarely<br>- Never<br><br>Worry affects daily work	p=0.02<br><br>4.79 (6.06)<br>12.43 (11.20)<br>3.38 (6.01)<br><br>p=0.02<br>2.69 (5.67)<br>5.61 (7.64)<br>10.63 (9.61)<br>18 (9.85)<br>5 (0)<br>7 (9.90)<br><br>p=0.02<br>6.74 (1.23)<br>3.20 (0.80)<br><br>p=0.002<br>10.04 (9.62)<br>4 (6.61)<br>5.13 (6.79)<br>1.82 (3.88)<br><br>p=0.0008<br>11.7 (10.6)<br>14.67 (11.60)<br>9.14 (6.94)<br>3 (5.33)<br><br>p=0.0003

		- Frequently	13 (10.37)
		<u>Multivariate association between the negative PCQ score and variables</u>	
		- Religion	p=0.55
		- Number of biopsies	p=0.01
		- Result last mammography	p=0.01
		- Worry	p=0.13
		- Affects humour	p=0.80
		- Affects daily work	p=0.19
		- Increases medical visits	p=0.14
		- Adherence	p=0.30
Gray, 2006	HADS; MHLCS; self-designed questionnaire (POSM)	HADS anxiety subscale	
		<u>Univariate analysis</u>	
		Age	
		Trial centre	p=0.010
		Marital status	p<0.001
		Employment status	p=0.019
		Training/Education	p<0.001
		Physical activity	p=0.001
		Ever had children	p=0.001
		Smoking status	p=0.001
		Index smear status	p<0.001
		Previous smear history	P=NS
		Ethnic group (White vs	P=NS
		Non-white)	P=NS

		<u>Multivariate analysis</u><br><br>Age group<br>- 20-29 years<br>- 30-39 years<br>- 40-49 years<br>- 50-59 years<br><br>Physical activity<br>- < Once/week<br>- 1 –3 times/week<br>- > 3 times/week<br><br>Ever had children<br>- No<br>- Yes<br><br>Smoking status<br>- Never smoker<br>- Ex-smoker<br>- Current smoker	 **OR (95% CI), p-value**<br><br>p=0.031<br>1.00<br>0.97 (0.78 – 1.22)<br>0.85 (0.66 – 1.10)<br>0.68 (0.48 – 0.97)<br><br>P=NR<br>1.00<br>1.13 (0.91 – 1.40)<br>1.52 (1.26 – 1.85)<br><br>p=0.025<br>1.00<br>1.26 (1.03 – 1.55)<br><br>p<0.001<br>1.00<br>1.22 (0.97 – 1.54)<br>1.52 (1.26 – 1.84)
Hilal, 2017	STAI	Multivariate Analyses<br><br><u>Linear</u><br>Study group<br>Study center<br>Age<br>BMI<br>Parity<br>Allergies<br>Smoking<br>Education level	 <br><br>P=NS<br>p=0.028<br>P=NS<br>p=0.033<br>P=NS<br>P=NS<br>p=0.025<br>P=NS

		Logistic<br>Study group<br>Study center<br>Age<br>BMI<br>Parity<br>Allergies<br>Smoking<br>Education level	 P=NS<br>P=NS<br>P=NS<br>P=NS<br>P=NS<br>P=NS<br>p=0.029<br>P=NS
Kola, 2012	STAI; PANAS; MBSS	Multiple logistic regression<br><br>Age<br>Single vs married<br>No children vs children<br>Less than tertiary vs tertiary education<br>All other smear grades vs high grade<br>Waiting time<br>Trait anxiety<br>Fear of minor pain<br>Monitoring style<br>Knowledge<br>Expectation of pain	 <br>P=NS<br>$p<0.05$<br>$p<0.05$<br>P=NS<br><br>P=NS<br><br>$p<0.05$<br>$p<0.01$<br>$p<0.05$<br>$p<0.01$<br>$p<0.05$<br>$p<0.01$
Liao, 2008	Modified version of the Mishel Uncertainty in Illness Scale; STAI	Predictive Factors for Uncertainty at 3 times (reference in parentheses)<br>Simple linear	

		regression<br><br><u>Time 1: notice of biopsy</u><br>- Marital status (not married)<br>- Education (junior high and under)<br>- Age (under 50)<br>- Religious status (nil)<br>- Family history of benign breast tumour (no)<br>- Regular breast self-examination (no)<br>- Self-perceived probability of breast cancer diagnosis (<50%)<br><br><u>Time 2: before biopsy</u><br>- Marital status (not married)<br>- Education (junior high and under)<br>- Age (under 50)<br>- Religious status (nil)<br>- Family history of benign breast tumour (no)<br>- Self-perceived probability of breast cancer diagnosis	 <br><br><br>p=0.030<br><br>P=NS<br><br>P=NS<br>p=0.004<br>P=NS<br><br><br>P=NS<br><br>P=NS<br><br><br><br><br><br>P=NS<br><br>P=NS<br><br>P=NS<br>p=0.004<br>p=0.043<br><br><br>p=0.013

		(<50%)<br><br><u>Time 3: after diagnosis</u><br>- Marital status (not married)<br>- Education (junior high and under)<br>- Age (under 50)<br>- Religious status (nil)<br>- Family history of benign breast tumour (no)<br>- Self-perceived probability of breast cancer diagnosis (<50%)<br>- Biopsy result (benign)	 <br><br>P=NS<br><br>P=NS<br><br>P=NS<br>P=NS<br>P=NS<br><br><br>P=NS<br><br><br><br>p=0.000
Maissi, 2004	STAI-6;<br>GHQ-12;<br>self-designed questionnaire	Linear multiple regression for predictors of<br><u>I. Anxiety</u><br>- Age (younger women vs older women)<br>- Perceived risk of cervical cancer<br>- Not knowing meaning of smear result<br><br><u>II. Distress and concern</u><br>- Perceived risk of developing cervical	 <br><br><br>$\beta = -0.11$, $P = 0.033$<br><br>$\beta = 0.17$, $P < 0.001$<br><br>$\beta = 0.17$, $P = 0.001$<br><br><br><br>Distress: $\beta = 0.20$, $P < 0.001$<br>Concern $\beta = 0.24$, $P < 0.001$

		cancer<br><br>- Not knowing the meaning of the result	 Distress: $\beta = 0.15$, $P < 0.001$<br>Concern: $\beta = 0.24$, $P < 0.001$
Medd, 2005	Self-designed questionnaire	Men's experiences during needle biopsy of the prostate<br><br><u>Anxiety surrounding testing</u><br>- Waiting for the result<br>- Fear of the result<br>- Not knowing what will happen during test<br>- Not knowing what will happen after test<br>- Feeling helpless during test<br>- Worry about waiting for the test<br><br><u>Procedure related</u><br>- Discomfort during test<br>- Pain during the test<br>- Thought of the test<br>- Fear about coping with the test<br>- Feeling embarrassed during test<br>- Fear of needles	**% ratings ≥7/10 (where 0 =no trouble at all) and 10 =worst trouble they could imagine)**<br><br><br><br><br>32%<br>32%<br>13%<br><br>23%<br><br>13%<br><br>3%<br><br><br><br>32%<br>29%<br>16%<br>6%<br><br>16%<br><br>16%<br>10%

		- Fear of infection after test<br>- Feeling undignified during test<br>- Fear of bleed after test<br>- Feeling exposed during test<br>- Worry about opening bowels during test<br>- Worry about passing urine during test<br>- Feeling violated during test<br>- Worry about not being able to see what's happening during test<br>- Worry about the sounds of the test<br>- Fear of bleeding during test	 13%<br><br>0%<br><br>13%<br><br>6%<br><br>6%<br><br>10%<br><br>3%<br><br><br>0%<br><br>0%
O'Connor, 2016	HADS-anxiety;<br>POSM	<u>Multivariate analysis of predictors of anxiety over 12 months post-colposcopy</u><br><br>Nationality<br>- Other vs Irish<br><br>Private health insurance<br>- No vs yes	**OR (95% confidence intervals), p-value**<br><br><br><br><br><br>p=0.020<br>2.13 (1.13–4.01)<br><br>p=0.006<br><br>1.84 (1.20–2.84)

		History of depression<br>- Yes vs no<br><br>Satisfaction with life<br>Per unit increase<br><br><br><br>Multivariate analysis of predictors of worries about future fertility over 12 months post-colposcopy<br>Age<br>- 30–40 years vs <30 years<br>- >40 years <30 years<br><br>Currently pregnant<br>- No vs yes<br><br>Smoking status<br>- Past smoker vs current smoker<br>- Never smoked vs current smoker<br><br>Multivariate analysis of predictors of worries about cervical cancer over 12 months post-colposcopy<br>Private health insurance	p<0.001<br>2.33 (1.51–3.60)<br><br>p<0.001<br>0.67 (0.59–0.76)<br><br><br><br><br><br><br><br><br>p=0.002<br>1.26 (0.81–1.96)<br><br>0.18 (0.06–0.51)<br><br>p=0.003<br>4.17 (1.61–10.81)<br><br>p=0.012<br>0.49 (0.28–0.86)<br><br>0.50 (0.30–0.83)<br><br><br><br><br><br><br><br>p=0.002

		- No vs yes<br><br>Smoking status<br>- Past smoker vs current smoker<br>- Never smoked vs current smoker<br><br>Satisfaction with life<br>- Per unit increase<br><br>Perceived severity of colposcopy exam<br>- Per unit increase	1.80 (1.25–2.61)<br><br>p=0.012<br>0.52 (0.33–0.80)<br><br>0.74 (0.48–1.15)<br><br><br>p=0.006<br>0.88 (0.80–0.96)<br><br>p<0.001<br><br>1.84 (1.45–2.33)
Wiggins, 2017	IES; PCOS	Multivariate analyses examining factors associated with IES Scores over time<br><br><u>IES-intrusion</u><br>Age<br><br>Education<br>- Baseline<br>- 4-month<br><br># previous TVS tests<br>- Baseline<br>- 4-month<br><br>Hx of abnormal TVS<br>- Baseline	**Estimated mean ratio (95% CI), p-value**<br><br><br><br><br><br>P=NS<br><br>p=0.014<br>0.93 (0.88–0.99), p<0.05<br>0.91 (0.85–0.98), p<0.01<br><br>p=0.021<br>1.00 (0.97–1.04), p=NS<br>0.94 (0.90–0.99), p<0.05<br><br>p=0.021<br>0.55 (0.35–0.87), p<0.01

		- 4-month	0.97 (0.55–1.70), P=NS
		Optimism	p<0.001
		- Baseline	0.92 (0.88–0.96), p<0.001
		- 4-month	0.95 (0.90–1.00), p<0.05
		Social support	P=NS
		Social constraint	p<0.001
		- Baseline	1.06 (1.04–1.08), p<0.001
		- 4-month	1.11 (1.08–1.14), p<0.001
		OC Family Hx in FDR	P=NS
		IES-Avoidance	
		Age	P=NS
		Education	p=0.007
		- Baseline	0.96 (0.91–1.01), P=NS
		- 4-month	0.89 (0.83–0.96), p=0.007
		# previous TVS test	p=0.013
		- Baseline	1.00 (0.97–1.04), P=NS
		- 4-month	0.93 (0.88–0.98), p<0.01
		Optimism	p=0.014
		- Baseline	0.94 (0.91–0.98), p<0.01
		- 4-month	0.98 (0.93–1.03), P=NS
		Social support	P=NS
		Social constraint	p<0.001

		- Baseline	1.06 (1.04–1.08), p<0.001		
		- 4-month	1.14 (1.11–1.16)p<0.001		
		OC family history in FDR	p=0.012		
		- Baseline	2.15 (1.04–4.42), p<0.05		
		- 4-month	0.64 (0.23–1.73) p=NS		
Wiggins, 2019	SF-12; IES	Multivariate association between variables and OC-specific distress trajectory membership	Medium-decreasing vs no distress<br>OLR (95% CI)	High-decreasing vs no distress<br>OLR (95% CI)	High-decreasing vs medium-decreasing<br>OLR (95% CI)
			P=NS	P=NS	P=NS
		Age			
		Years of education	P=NS	P=NS	P=NS
		- Avoidance	P=NS	0.85 (0.75, 0.97), p<0.05	P=NS
		- Intrusion			
		# previous routine TVS	P=NS	P=NS	P=NS
		- Avoidance	P=NS	P=NS	0.91 (0.84, 0.99), p<0.05
		- Intrusion			
		No history of abnormal			
		TVS	2.26 (1.08, 4.75), p<0.05	P=NS	P=NS
		- Avoidance	2.60 (1.23, 5.52), p<0.05	P=NS	P=NS
		- Intrusion			
		Family history of OC in			
		FDR	2.53 (1.34, 4.81), p<0.01	2.55 (1.10, 5.92), p<0.05	P=NS
		- Avoidance	2.91 (1.52, 5.60), p<0.05	5.41(2.39, 12.26),p<0.01	P=NS
		- Intrusion			

		Physical functioning	P=NS	P=NS	P=NS
		- Avoidance	1.01 (1.00, 1.03), p<0.05	P=NS	P=NS
		- Intrusion			
		Optimism	P=NS	0.89 (0.80, 0.99), p<0.05	P=NS
		- Avoidance	0.91 (0.84, 0.99), p<0.05	0.77(0.69, 0.86),p<0.001	0.84(0.77, 0.92), p<0.001
		- Intrusion			
		Monitoring	1.24 (1.05, 1.46), p<0.05	P=NS	P=NS
		- Avoidance	P=NS	1.39 (1.13, 1.70), p<0.05	P=NS
		- Intrusion			
		Social support	P=NS	P=NS	P=NS
		- Avoidance	P=NS	1.12 (1.05, 1.20), p<0.05	1.10 (1.04, 1.17), p<0.05
		- Intrusion			
		Social constraint	1.16(1.09, 1.24), p<0.001	1.29(1.21, 1.39),p<0.001	1.11 (1.07, 1.16),p<0.001
		- Avoidance	1.15 (1.09, 1.21),p<0.001	1.27(1.19, 1.36),p<0.001	1.11 (1.06, 1.16),p<0.001
		- Intrusion			

†Abbreviations:

Measurement tools include COS-BC - Consequences of Screening in Breast Cancer; GHQ-12 – 12 item General Health Questionnaire; HADS – Hospital Anxiety and Depression Scale; HIP – HPV Impact Profile; IES – Impact of Event Scale; MBSS – Miller Behavioural Style Scale; MHLCS – Multi-dimensional Health Locus of Control Scale; MUIS - Mishel Uncertainty in Illness Scale; PANAS – Positive and Negative Affect Schedule; POSM - Process Outcome Specific Measure; SF – Short Form Survey; STAI - State-Trait Anxiety Inventory.

Other include OR – odds ratio; CI – confidence interval; SD – standard deviation; NS – not significant; NR – not reported; OLR – odds-like ratio; FDR – first degree relative; TVS – transvaginal ultrasonography

Table S3. Cross Sectional Study Results: Variables associated with psychological morbidity
