# Supplementary material for: Evaluating the Harms of Cancer Testing—A Systematic Review of the Adverse Psychological Correlates of Testing for Cancer and the Effectiveness of Interventions to Mitigate These
Source: Cancers (Basel). 2023 Jun 25;15(13):3335. doi: 10.3390/cancers15133335 (PMC10340425; doi:10.3390/cancers15133335)
Supplement: Supplementary file 1 [file cancers-15-03335-s001.zip › File S6. Search strategy.pdf]

The search strategy used for MEDLINE (Ovid) database is provided below.

1. (adult).ti,ab
2. (screen*).ti,ab
3. (test*).ti,ab
4. (detect*).ti,ab
5. (investigat*).ti,ab
6. (diagnos*).ti,ab
7. (biops*).ti,ab
8. (mass*).ti,ab
9. (tumo*).ti,ab
10. (neoplas*).ti,ab
11. (malignan*).ti,ab
12. (cancer*).ti,ab
13. (carcinoma*).ti,ab"
14. (anxiety) .ti,ab
15. (anxious) .ti,ab
16. (quality of life) .ti,ab
17. (satisf*).ti,ab
18. (worry).ti,ab
19. (nervous).ti,ab
20. (concern*).ti,ab
21. (fear*).ti,ab
22. (apprehens*).ti,ab
23. (psychologic*).ti,ab
24. (psychosocial).ti,ab
25. (affect*).ti,ab
26. (distress*).ti,ab
27. (stress*).ti,ab
28. (emotion*).ti,ab

1 AND (2 OR 3 OR 4 OR 5 OR 6 OR 7) AND (8 OR 9 OR 10 OR 11 OR 12 OR 13) AND (14 OR 15 OR 16 OR 17 OR 18 OR 19 OR 20 OR 21 OR 22 OR 23 OR 24 OR 25 OR 26 OR 27 OR 28)
