# Supplementary figures and images for: Evaluating the Harms of Cancer Testing—A Systematic Review of the Adverse Psychological Correlates of Testing for Cancer and the Effectiveness of Interventions to Mitigate These
Source: Cancers (Basel). 2023 Jun 25;15(13):3335. doi: 10.3390/cancers15133335 (PMC10340425; doi:10.3390/cancers15133335)

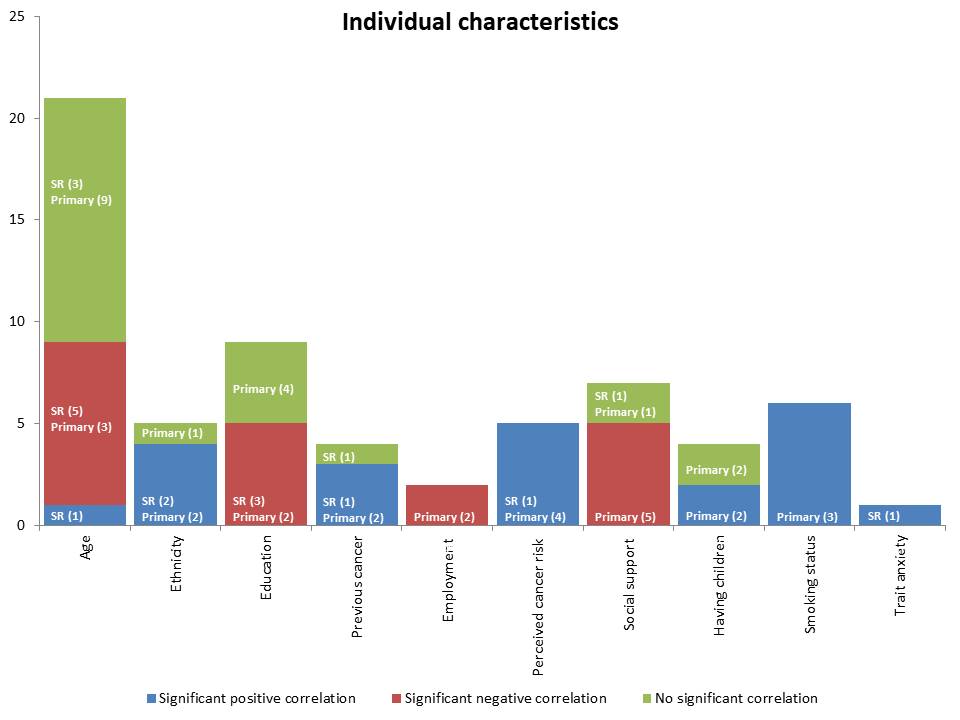

Supplement: Supplementary file 1 [file cancers-15-03335-s001.zip › File S4. Illustration of results for question 1 (individual characteristics).JPG]

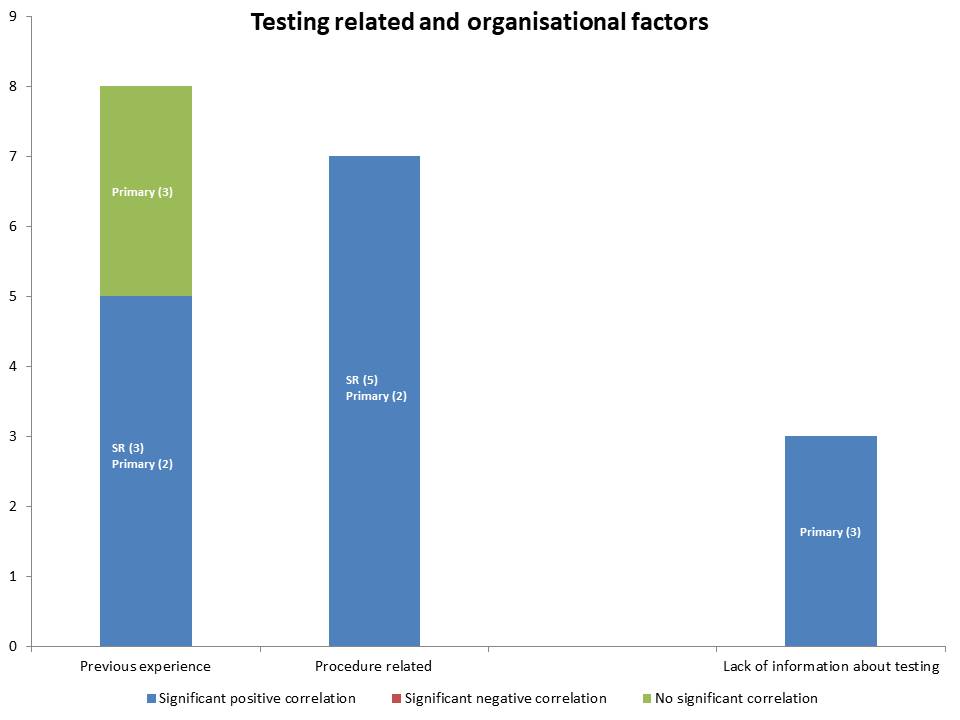

Supplement: Supplementary file 1 [file cancers-15-03335-s001.zip › File S5. Illustration of results for question 1 (testing related and organisational factors).JPG]
